# Evaluating heterogeneity in MDA‐5+ dermatomyositis through cluster analyses

**DOI:** 10.1111/jdv.70303

**Published:** 2026-01-12

**Authors:** David R. Pearson

**Affiliations:** ^1^ Department of Dermatology University of Minnesota Minneapolis Minnesota USA

Dermatomyositis is a multisystem autoimmune disease that may affect the skin, musculoskeletal system, respiratory system and others. There is significant heterogeneity in clinical manifestations, prognosis and response to treatment. Despite limitations, clinical phenotypes are often associated with individual myositis‐specific antibodies (MSA). Patients with MSA directed against Melanoma Differentiation Antigen‐5 (MDA‐5) may develop rapidly progressive interstitial lung disease (RP‐ILD), the leading cause of death in this subset, with a mortality risk that may exceed 80% within 3 months of diagnosis.^1^, ^2^

However, significant heterogeneity exists among patients with MDA‐5 autoantibodies. Prior studies of Chinese^1^ and predominantly northern European and Black^2^ cohorts have employed unsupervised cluster analyses to identify three similar clinical phenotypes: (1) Patients who develop RP‐ILD and experience high mortality; other notable features included mechanic's hands, elevated inflammatory markers, and lower likelihood of proximal myositis (32% vs. 18% of patients in each study, respectively). (2) Patients with lower rates of RP‐ILD and more typical cutaneous and proximal muscle symptoms (27% vs. 55%). (3) Patients at lower risk of RP‐ILD but demonstrable vasculopathy, with cutaneous ulcers, Raynaud's phenomenon, and proximal myositis (41% vs. 27%).

He et al.'s study^3^ is an important addition to this growing literature because it builds upon methodology and rigour. It also identifies three phenotypes, though they differ somewhat from those previously described. Utilizing unsupervised cluster analysis followed by a robust sensitivity analysis, they define: (1) a similar high‐risk RP‐ILD cohort; (2) a dermato‐rheumatologic cohort with typical skin and musculoskeletal involvement and lower risk of RP‐ILD and (3) a novel, low‐risk group with the lowest rate of RP‐ILD and dermato‐rheumatologic manifestations. The difference in 3‐month all‐cause mortality between these groups is striking: 88.7% vs. 4.3% vs. 2.4%, respectively, and mirrors rates of RP‐ILD (84.9% vs. 18.1% vs. 17.1%).

An important strength of this study is its inclusion criterion of radiographically demonstrated ILD by high‐resolution computed tomography. As He et al. discusses, these features are critical in the early diagnosis of RP‐ILD and carry important prognostic implications. While reduction in dimensionality through application of principal component (PC) analysis allowed for incorporation of these data into the cluster analysis, and functions as a critical component of the model, it does pose a challenge for clinicians: how do we easily calculate the PC1 score in the office for our patients? Further refinement of this model, hopefully without loss of integrity, may prove a highly useful bedside tool.

The heterogeneity of patients with MDA‐5 autoantibodies, and dermatomyositis in general, is further complicated: how well can we as clinicians rely on commercially available MSA testing? Lack of standardization among commercial antibody panels, lack of widespread availability of gold standard immunoprecipitation testing for MSA, and challenge in identifying patients with high pretest probability all influence the positive and negative predictive values of these tests. In comparison to immunoprecipitation, line immunoassay (LIA) and enzyme‐linked immunosorbent assays (ELISA) do not perform equally across MSAs; a recent meta‐analysis^4^ found better agreement with immunoprecipitation for MDA‐5 autoantibodies (LIA, sensitivity 82.8%, specificity 96.3%; ELISA, sensitivity 95.7%, specificity 99.7%) versus transcriptional intermediary factor 1‐γ (TIF1γ) autoantibodies (LIA, sensitivity 63.8%, specificity 96.5%). A lack of positive antibodies does not eliminate risk of severe outcomes; Chen et al.^5^ found an incidence of ILD of 46.8%; 25% of these patients demonstrated RP‐ILD.

In summary, phenotypic differentiation of dermatomyositis is crucial in identifying high‐risk patients, and He et al. expands the literature in defining these groups. Accurate modelling and biomarkers will allow us to better care for high‐risk patients, and above all, we must remain vigilant for RP‐ILD in all our dermatomyositis patients, even those who do not have positive MSAs.

## CONFLICT OF INTEREST STATEMENT

Grants or contracts: Daiichi Sankyo Company Ltd., EMD Serono Inc., Nkarta Inc., Priovant Therapeutics. Consulting: AstraZeneca plc, Biogen Inc., Merck & Co Inc., Pfizer Inc. Board membership: Minnesota Dermatological Society. Steering committee membership: EMD Serono Inc.

## Data Availability

Data sharing is not applicable to this article as no datasets were generated or analysed during the current study.
